# pH–Dependent Luminescence of Self–Assembly Tb^3+^ Complexes with Photosensitizing Units

**DOI:** 10.1002/chem.202502378

**Published:** 2025-09-19

**Authors:** Eiko Mieda, Tatsuya Watanabe, Ryusei Morita, Hiroyuki Miyake, Satoshi Shinoda

**Affiliations:** ^1^ Department of Chemistry Graduate School of Science Osaka Metropolitan University 3–3–138 Sugimoto Sumiyoshi–ku Osaka 558–8585 Japan

**Keywords:** amphiphilic molecule, lanthanide complex, pH–dependent luminescence, self‐assembly, sensitized luminescence

## Abstract

Luminescent metal complexes in aqueous solution have been attracting substantial interest as chemical and biological sensors. In this study, aiming to develop lanthanide complexes that exhibit bright luminescence in aqueous media, we designed and synthesized new amphiphilic ligands based on ethylenediamine with two aromatic groups and two cholesteryl groups, which act as photosensitizers and hydrophobic moieties, respectively. In particular, the Tb^3+^ complex of a pyridyl‐substituted ligand showed an intense green emission that stems from the efficient energy transfer from the excited pyridyl groups to the Tb center in ethanol. In aqueous media, the Tb^3+^ complexes self‐assembled into nanoparticles with diameters of around 40 nm, which show pH–dependent luminescence in the pH range of 6.0–8.5 ascribable to protonation of the coordinated pyridyl groups. These findings demonstrate the potential of aggregates of photosensitized lanthanide complexes as sensors under weakly alkaline conditions.

## Graphical abstract

An amphiphilic lanthanide complex with photosensitizer units forms stable self‐assemblies in aqueous ethanol (20 wt.% EtOH). The pyridyl group effectively works as both a photoantenna and a proton acceptor. Self‐assemblies of the Tb^3+^ complex exhibit pH–dependent luminescence under weakly basic conditions, rendering it promising for biological applications.

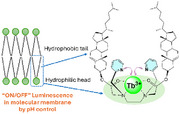

## 1 Introduction

Luminescent metal complexes are essential in a wide range of scientific and technological fields owing to their unique properties.^[^ ^1^ ^]^ In particular, luminescent lanthanide complexes have attracted considerable interest as luminescent chemical sensors, biosensors, security ink, and molecular thermometers.^[^ ^2^ ^]^ Luminescent trivalent lanthanide ions (Ln^3+^) are characterized by long‐lived excited states and a narrow emission band width; however, the direct excitation of Ln^3+^ to the emitting state via f–f transitions is Laporte‐forbidden, and the absorption coefficient is generally small. Therefore, sensitization of the Ln^3+^ center via energy transfer by introducing an “antenna site” is required.^[^ ^3^, ^4^, ^5^ ^]^ In general, aromatic chelating ligands such as bipyridine, phenanthroline, and β‐diketonate can act as effective photosensitizers.^[^ ^6^, ^7^ ^]^ The luminescence intensity of Ln^3+^ complexes varies with changes in the coordination environment around the Ln^3+^ center. For instance, their luminescence intensity significantly decreases in aqueous solution owing to the direct coordination of water molecules to Ln^3+^, which converts the excited‐state energy of Ln^3+^ into O–H vibrations, leading to its deactivation.^[^ ^3^ ^]^ Thus, chelating ligands are required not only to construct highly stable coordination compounds but also to prevent water coordination. Many water‐soluble Ln^3+^ complexes with hydrophilic ligands have been reported.^[^ ^3^, ^6^, ^7^, ^8^, ^9^, ^10^, ^11^, ^12^, ^13^, ^14^ ^]^ For example, Silva et al. have reported water‐soluble Eu^3+^ complexes bearing ethylenediaminetetraacetic acid (EDTA) and β‐diketone ligands, which exhibit strong luminescence in aqueous media.^[^ ^12^ ^]^ Hasegawa et al. have synthesized a water‐soluble luminescent Eu^3+^ complex containing an ethylenediamine‐based bowl‐shaped ligand,^[^ ^13^, ^14^ ^]^ which forms a helical hexadentate coordination structure with two carboxylate groups, one coordinating to the metal center to prevent water coordination and the other providing solubility in water.

To achieve strong luminescence in aqueous media, molecular self‐assembly using amphiphilic molecules has emerged as a promising method owing to the unique properties of self‐assembled supramolecules, which differ from those of single molecules. Some examples on the self‐assembly of Ln^3+^ complexes have been reported. Kimizuka et al. have synthesized nanoparticles of self‐assembled Ln^3+^ complexes using a hydrophobic longalkylchain ligand^[^ ^15^, ^16^ ^]^ and ionic lipids^[^ ^17^ ^]^ in aqueous ethanol. Hasegawa et al. have used micelle reaction techniques to fabricate strongly luminescent nanoparticles composed of Ln^3+^ coordination polymers^[^ ^18^ ^]^ and have also reported that their particle size can be changed by varying the length of the alkyl chain of the surfactant in water.^[^ ^19^ ^]^ We have reported self‐assembled amphiphilic Eu^3+^ complexes that are based on the ligand 1,4,7,10‐tetraazacyclododecane‐1,4,7,10‐tetraacetic acid (DOTA), which exhibit bright and long‐lived luminescence in aqueous ethanol (20 wt% EtOH). The Eu^3+^ complex assemblies to form round particles with a size of 10–20 nm estimated by transmission electron microscopy (TEM) image under consideration of a bilayer membrane or oblate micelle. By using anion–displacement techniques, the Eu^3+^–complex assembly allowed the selective sensing of hydrophobic anions such as perchlorate and hexafluorophosphate by the naked eye detection.^[^ ^20^ ^]^

**Scheme 1:**
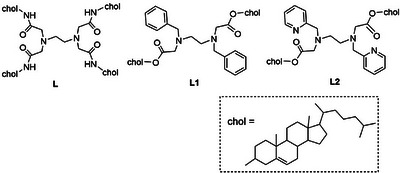
Structures of cholesterol‐substituted ligands.

Ln^3+^ complexes with coordination sites where ligand–exchange reactions easily occur exhibit luminescence spectral changes in response to pH changes and guest binding.^[^ ^9^, ^10^, ^21^ ^]^ Recently, we have synthesized an EDTA ligand that carries four cholesteryl groups (**L**)^[^ ^22^ ^]^ (Scheme 1). The hexadentate ligand **L** forms complexes that contain exchangeable water coordination sites that can accommodate external guests. In particular, ligand **L** forms stable 1:1 complex with Tb^3+^, which exhibit brighter luminescence in aqueous media than in EtOH by forming colloidal nanoparticles. **L**‐Tb can also form ternary complexes in combination with several anionic guest species, thus enabling the effective sensitization of Tb^3+^ luminescence. However, such guest species are limited to hydrophobic species, which are miscible with the hydrophobic membrane. In addition, this sensitizing strategy for Ln^3+^ luminescence in water is not fully effective in energy‐transfer processes due to the external addition of sensitizing substrates, and due to the molecular recognition of various further guest species.

Luminescent Ln^3+^ complexes can act as pH indicators in bio tools and materials.^[^ ^9^, ^10^, ^23^, ^24^ ^]^ For example, Parker et al. have reported pH−dependent Ln^3+^ luminescence via intramolecular switching of the hydration state,^[^ ^25^ ^]^ whereby the deprotonation of an NH proton of arylsulfonamide is responsible for the on–off switching of the luminescence. Wei et al. have reported the pH–induced control over the turn−on emission in water‐soluble Eu^3+^ complexes with unique UV−light stability.^[^ ^26^ ^]^ Recently, Shi et al. have reported reversible pH−responsive changes in the luminescence of Tb^3+^ and Eu^3+^ complexes with a 2,6‐pyridinedicarboxylic acid ligand both in aqueous solution and in the solid state.^[^ ^27^ ^]^ In addition, pH–dependent luminescence has been reported for a MOF‐type Ln^3+^ complex,^[^ ^28^, ^29^ ^]^ a mesoporous supported material,^[^ ^30^ ^]^ and a polymeric hydrogel.^[^ ^31^ ^]^ Thus, the development of materials for pH–sensing applications based on these structural changes has garnered intense attention. In particular, stimuli–responsive materials that exhibit “off–on–off” or “on–off–on” luminescence switching are interesting to design enzymes and logic gate mimics.^[^ ^32^, ^33^, ^34^ ^]^ In many of these materials, the donor part of the ligand, which works as a photoantenna, reacts with a proton to induce a structural change or is displaced from the metal center by coordinating water, causing luminescence intensity changes. Alternatively, these reactions also proceed on the material surface in a heterogeneous manner using supported Ln^3+^ complexes. If the molecular behavior of the homogeneous solution system can be demonstrated in the same function in the molecular assembly of the heterogeneous system without affecting the shape or thickness of the molecular membranes, it can be an advantage as a bulk material.

In this study, ligands **L1** and **L2** were designed and synthesized by replacing two cholesteryl groups in **L** with aromatic groups as photosensitizers (Scheme 1). Their complexation studies with Lu^3+^ were investigated using NMR spectroscopy. Self‐assembly behavior and luminescence properties of their Tb^3+^ complex in aqueous ethanol solution were assessed by dynamic light scattering (DLS) studies and photoluminescence measurements. The pH dependence of the luminescence intensity of self‐assembled Tb^3+^ complexes was revealed by a pH–titration study.

## 2 Results and Discussion

### 2.1 Synthesis of Cholesterol‐substituted Ligands

**Scheme 2:**
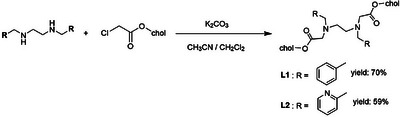
Synthesis of benzyl‐ and pyridylmethyl‐substituted ligands **L1** and **L2**.

Doubly cholesterol‐substituted ligand **L1**, which also contains two benzyl substituents, was synthesized by reacting 2 eq. of cholesten chloroacetate^[^ ^35^ ^]^ with *N*,*N*″–bis(phenylmethyl)‐1,2‐ethanediamine in the presence of an excess of K_2_CO_3_ in CH_3_CN /CH_2_Cl_2_ (Scheme 2). Similarly, pyridyl type ligand **L2** was synthesized using *N*,*N*″–bis(2‐pyridylmethyl)‐1,2‐ethanediamine as a precursor.^[^ ^36^ ^]^

### 2.2 Complexation Studies via NMR Spectroscopy in Organic Solvents

**Figure 1:**
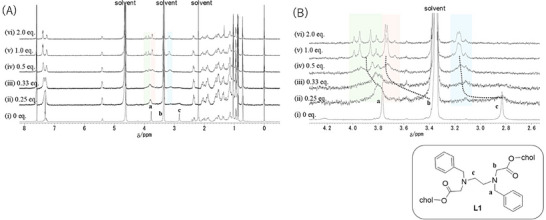
^1^H NMR spectral changes of **L1** upon addition of Lu(OTf)_3_ in CDCl_3_/CD_3_OD (1:1, v/v). [**L1**] = 1.0 mM, [Lu(OTf)_3_] = i) 0 mM, ii) 0.25 mM, iii) 0.50 mM, iv) 0.75 mM, v) 1.0 mM, and vi) 2.0 mM [A) full spectra; B) expanded region around the coordination sites].

EDTA‐type hexadentate ligand **L**, which bears four cholesteryl groups, forms stable 1:1 complexes with Ln^3+^ in solution.^[^ ^22^ ^]^ As benzyl‐substituted **L1** contains less donor atoms than **L**, its complexation stoichiometry might be different. To determine the complexation stoichiometry of **L1** with Ln^3+^ in organic solvents, the reaction of **L1** with increased amounts of lutetium trifluoromethanesulfonate (Lu(OTf)_3_) was monitored using ^1^H NMR spectroscopy in a mixture of CDCl_3_ and CD_3_OD (1:1, v/v) solvent (Figure 1(A) and (B)). Tb^3+^ ions contain unpaired electrons in degenerate f orbitals and are thus paramagnetic. In the NMR spectra of paramagnetic complexes, it is often difficult to accurately assign the peaks of the coordination sites and evaluate the reaction rate due to paramagnetic shifts. Therefore, the NMR experiments were carried out using Lu(OTf)_3_, which is diamagnetic.

The addition of 0.25 eq. of Lu(OTf)_3_ caused a considerable broadening of the signals related to the coordination sites of the ligand. Upon adding 0.33 eq. of Lu(OTf)_3_, the signals for the ethylene protons of the ligand backbone were downfield shifted from 2.8 to 3.2 ppm due to coordination to metal ion. When 0.5 eq. of Lu(OTf)_3_ was added, the signal of the benzyl protons split into two peaks. Upon increasing the amount of Lu(OTf)_3_ added from 0.5 to 1.0 eq., the two methylene proton signals (**a** and **b**) split into two doublets, which suggests that the complexation of **L1** with Lu^3+^ had a more static nature. (Figure 1, spectrum (iv) to (v)), Meanwhile, the signals from (v) to (vi) (L:M = 1:1 to 1:2) showed almost no change, indicating complexation in a 1:1 stoichiometry to give a static structure in solution.

**Figure 2:**
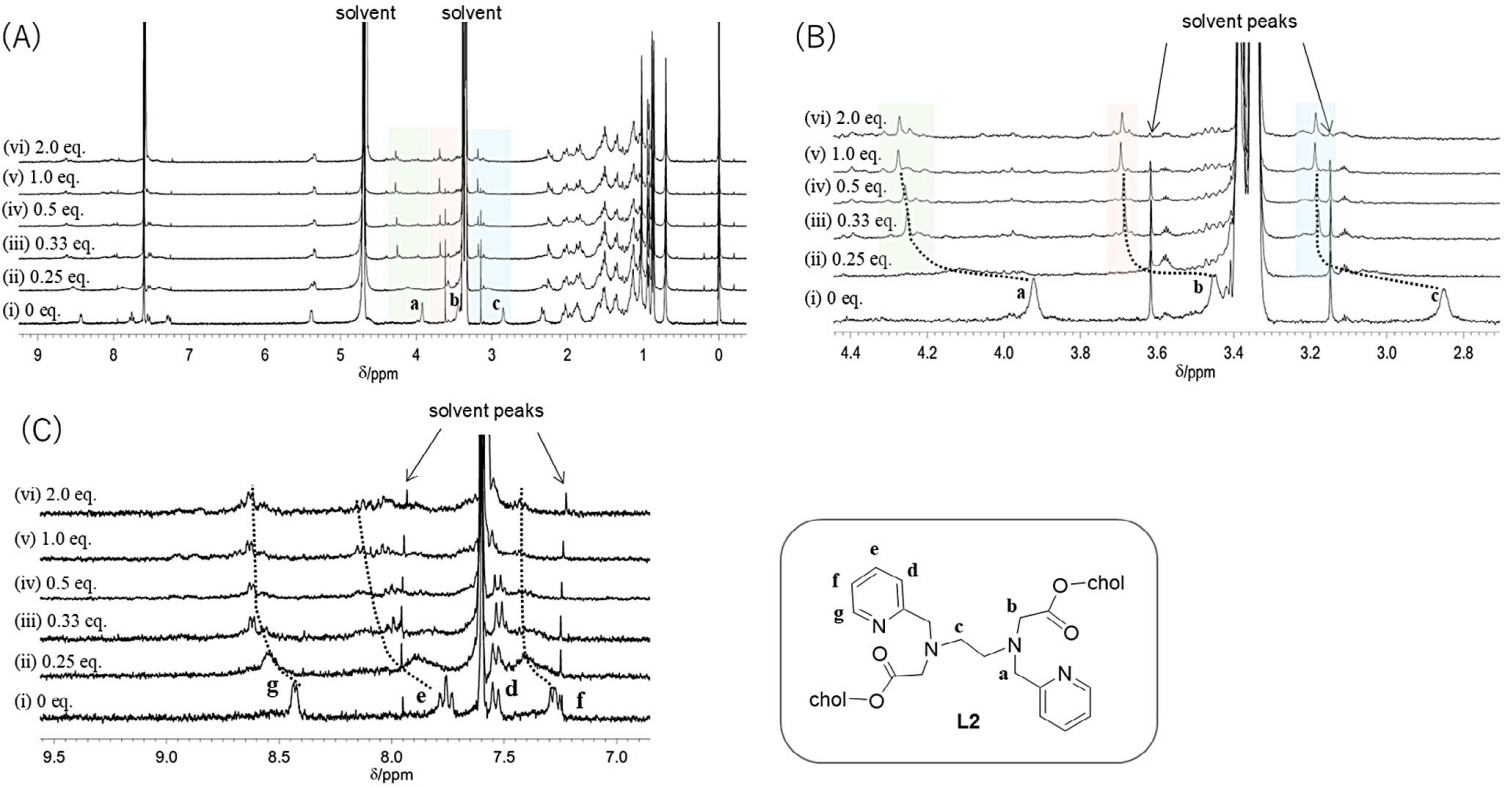
^1^H NMR spectral changes of **L2** upon addition of Lu(OTf)_3_ in CDCl_3_/CD_3_OD (1:1, v/v). [**L2**] = 1.0 mM, [Lu(OTf)_3_] = i) 0 mM, ii) 0.25 mM, iii) 0.50 mM, iv) 0.75 mM, v) 1.0 mM, and vi) 2.0 mM [A) full spectra; B) expanded region around the core sites; C) expanded region around the pyridine moieties].

We also carried out a titration study of **L2** with Lu^3+^ (Figure 2(A), (B) and (C)). Compared to **L1**, the titration change was simpler for **L2**, which can be attributed to the increase in coordination sites from four to six, thus facilitating the coordination with Lu^3+^. When 0.25 eq. of Lu^3+^ was added, the signals of free **L2** shifted and broadened. In particular, the methylene protons near the coordination sites (**a**, **b**, and **c**) disappeared (Figure 2(B), spectrum (ii)). The signals resolved from (ii) to (iii) (L:M = 1:0.25 to 1:0.33) upon increasing the **L2**: Lu^3+^ ratio, which suggests that isomers with different coordination geometries may exist.

Meanwhile, negligible changes were observed after adding 0.5 eq. of Lu(OTf)_3_ or more, suggesting the formation of a 1:1 complex. The peaks of the pyridine ring were also downfield shifted, and unidentified signals were observed in the low–field region when adding more than 1.0 eq. of Lu(OTf)_3_. This indicates that the coordination to Lu^3+^ affects the signals of the pyridine ring and results in the formation of a complicated mixture of compounds with different coordination geometries in solution.

### 2.3 Luminescence Properties of Tb^3+^ Complexes in EtOH

**Figure 3:**
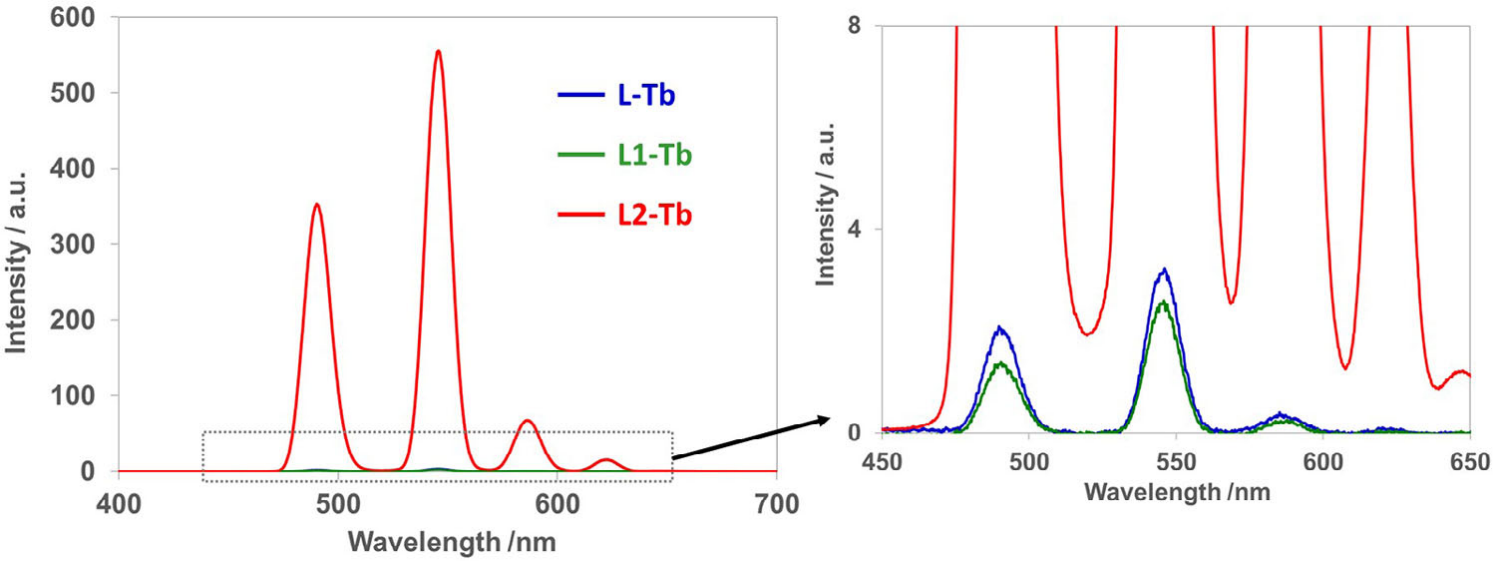
Luminescence spectra of **L**‐Tb, **L1**‐Tb, and **L2**‐Tb. [**L**‐Tb] = [**L1**‐Tb] = [**L2**‐Tb] = 2.0 × 10^−5^ M in EtOH (**L**‐Tb and **L1**‐Tb, *λ*_ex_ = 230 nm; **L2**Tb, *λ*_ex_ = 260 nm). Right: expanded region at 450–650 nm.

In EtOH, **L1** and **L2** showed absorption derived from their aromatic groups at 240 nm (abs = 0.051) and 260 nm (abs = 0.126), respectively (Figure S1). The **L**‐Tb was excited at the absorption band of the carbonyl group, and the **L1**‐Tb was excited at phenyl rings. The excitation wavelength was used at 230 nm. The **L2**‐Tb was excited at the pyridine ring using 260 nm excitation light. The luminescence spectra of the nonassembled Tb^3+^ complexes with **L** (**L**‐Tb), **L1** (**L1**‐Tb), and **L2** (**L2**‐Tb) in EtOH are shown in Figure 3. The strongest peak at 546 nm corresponds to the ^5^D_4_ → ^7^F_5_ transition. **L2**‐Tb showed the most intense luminescence because the pyridyl groups act as an effective photoantenna for the excitation of Tb^3+^.^[^ ^37^, ^38^, ^39^ ^]^ This is likely due to the energy level of **L2** being closer to the emissive energy level of the visible emitting Tb ions. Conversely, the luminescence intensity of **L1**‐Tb was as low as that of **L**‐Tb, which does not contain any photoantenna substituent; this can be interpreted in terms of the weak coordination ability of **L1** and/or the low stability of the complex.

### 2.4 Characterization of the Self–assembly via DLS Measurements

**Figure 4:**
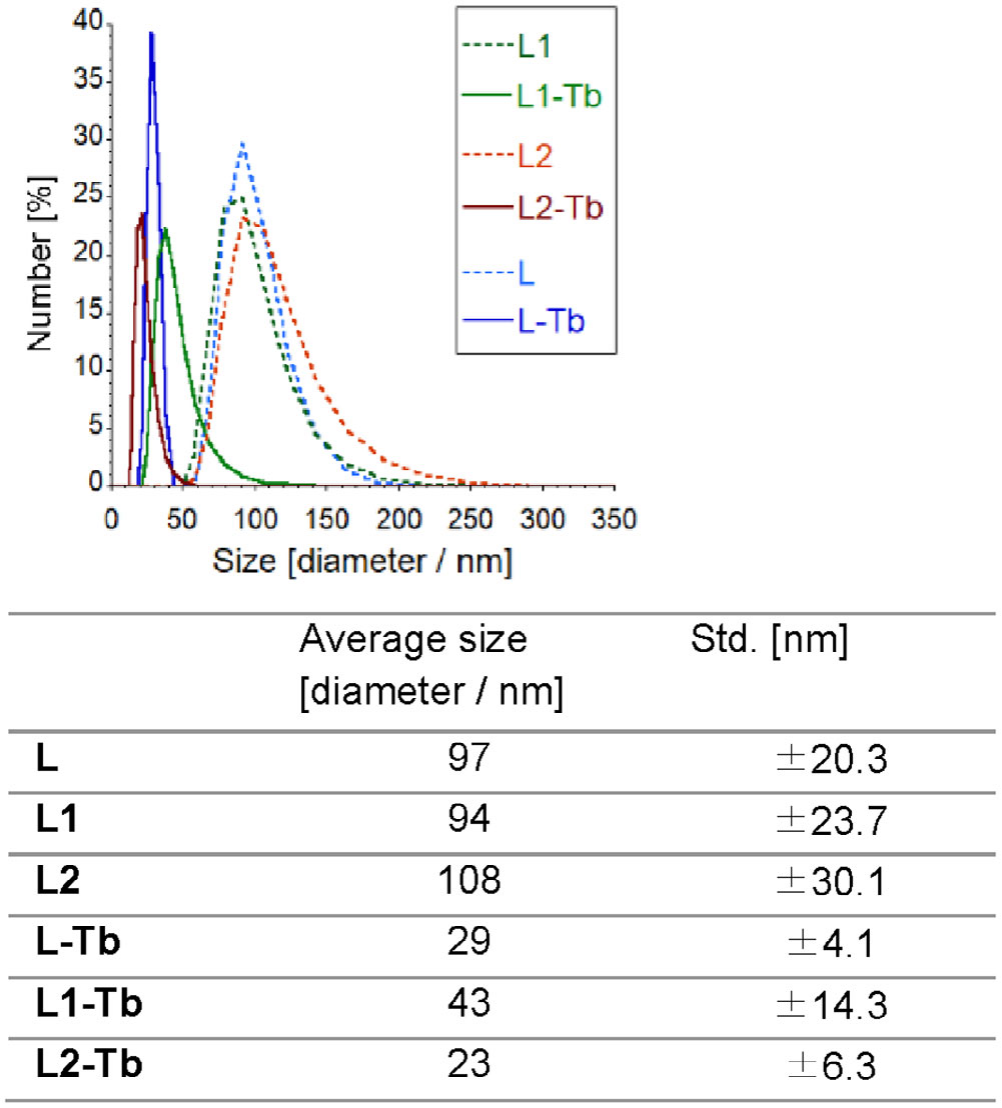
Particle size distribution plots of **L**, **L1**, **L2**, and their Tb^3+^ complexes estimated based on DLS measurements. The average size and standard deviations (Std.) are listed in the table. [**L**] = [**L1**] = [**L2**] = [**L**‐Tb] = [**L1**‐Tb] = [**L2**‐Tb] = 2.0 × 10^−5^ M; pH = 6.0; aqueous ethanol (20 wt.% EtOH).

Our previously reported amphiphilic Ln^3+^ complex and the **L** ligand bearing four cholesteryl groups form stable self‐assembled nanoparticles in aqueous ethanol solution.^[^ ^22^ ^]^ In the case of **L1** and **L2**, which contain two cholesteryl groups, weaker hydrophobic interactions can be expected compared to those of **L**. To confirm the self‐assembly behavior of the less hydrophobic systems with **L1** and **L2**, DLS measurements were performed in aqueous ethanol (20 wt.% EtOH) (Figure 4). **L1** and **L2** formed self‐assembled nanoparticles (diameter ≈ 100 nm) due to their amphiphilic nature in water stemming from the protonation of the ethylenediamine moiety. When adding an equimolar amount of terbium trifluoromethanesulfonate (Tb(OTf)_3_), the resulting Tb^3+^ complexes formed smaller self‐assembled nanoparticles (diameter ≈ 40 nm). The different size of the self‐assemblies may be due to conformational differences between the ligands and the complexes. Despite having a lower number of coordination sites, **L1**, **L2**, and their complexes form stable nanoparticles and show self‐assembly behavior similar to that of **L**.

### 2.5 Luminescence Properties of Tb^3+^ Complexes in Aqueous Media

**Figure 5:**
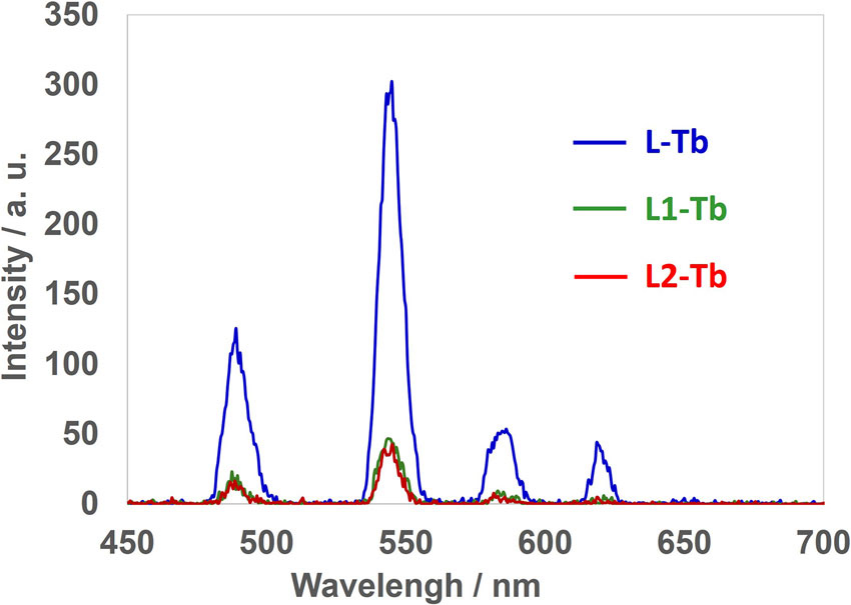
Luminescence spectra of **L**‐Tb, **L1**‐Tb, and **L2**‐Tb. [**L**‐Tb] = [**L1**‐Tb] = [**L2**‐Tb] = 2.0 × 10^−5^ M; buffer: 1.7 × 10^−3^ M bis‐tris (pH = 7.5; HCl) in aqueous ethanol (20 wt.% EtOH) (**L**‐Tb and **L1**‐Tb: *λ*_ex_ = 230 nm; **L2**‐Tb: *λ*_ex_ = 260 nm).

According to the DLS results, **L1**‐Tb and **L2**‐Tb exist as stable nanoparticles in aqueous media. Therefore, the luminescence properties of self‐assembled **L1**‐Tb and **L2**‐Tb were examined. The luminescence intensity considerably decreased compared to that of the self‐assemblies with **L** (Figure 5), suggesting that water coordinates easily to the metal center.

### 2.6 Effect of the pH Value on the Luminescence Properties

**Figure 6:**
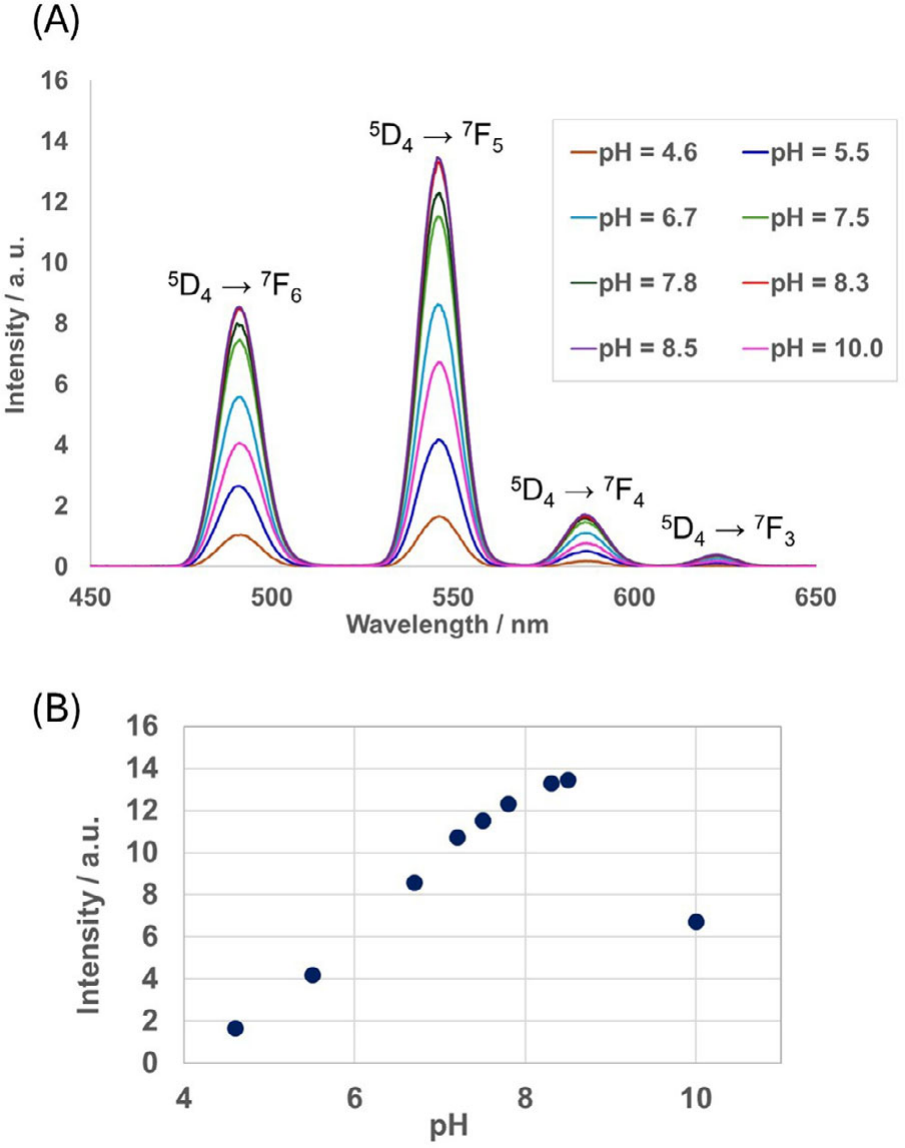
a) Spectral changes of self‐assembled **L2**‐Tb at varying pH values. b) Plot of the intensity at 546 nm as a function of the pH value. [**L2**‐Tb] = 1.0 × 10^−4^ M in aqueous ethanol (20 wt.% EtOH); excitation wavelength (*λ*_ex_) = 260 nm.

To explore the effect of the pH value on the luminescence properties of self‐assembled **L**‐Tb, **L1**‐Tb, and **L2**‐Tb, luminescence spectral measurements were conducted under varying pH conditions. The excitation wavelength of self‐assembled **L**‐Tb and **L1**‐Tb was 230 nm (abs = 0.007 and 0.051 for **L**‐Tb and **L1**‐Tb, respectively). For **L2**‐Tb, an excitation wavelength of 260 nm (abs = 0.126) was used. **L2** contains pyridyl groups that work as both protonation sites and coordination ligands for Ln^3+^. Thus, self‐assembled **L2**‐Tb was expected to exhibit pH–responsive luminescence. Figure 6(a) shows the luminescence spectra of self‐assembled **L2**‐Tb at pH values between 4 and 10. The change in the intensity at 546 nm (^5^D_4_ → ^7^F_5_) with varying pH value is shown in Figure 6(b). Below pH = 4.0, a very weak luminescence was observed, probably because the pyridyl groups undergo protonation. With increasing the pH from 4.6 to 8.5, the characteristic peaks of Tb^3+^ appear and the luminescence intensity gradually increases until reaching its maximum at pH = 8.5. Conversely, further increasing the pH value from 8.5 to 10.0 decreases the luminescence intensity. This luminescence quenching may be due to the dissociation of Tb^3+^ to form hydroxide species. It was found that the change of luminescent intensity with pH of the assemblies was slightly slower than reported pH response of the single molecule in solution.^[^ ^23^ ^]^ To form the assemblies, the reaction rate inside the membrane differs from the reaction rate on the surface, which may indicate a slower response than a free single molecule.

**Scheme 3:**
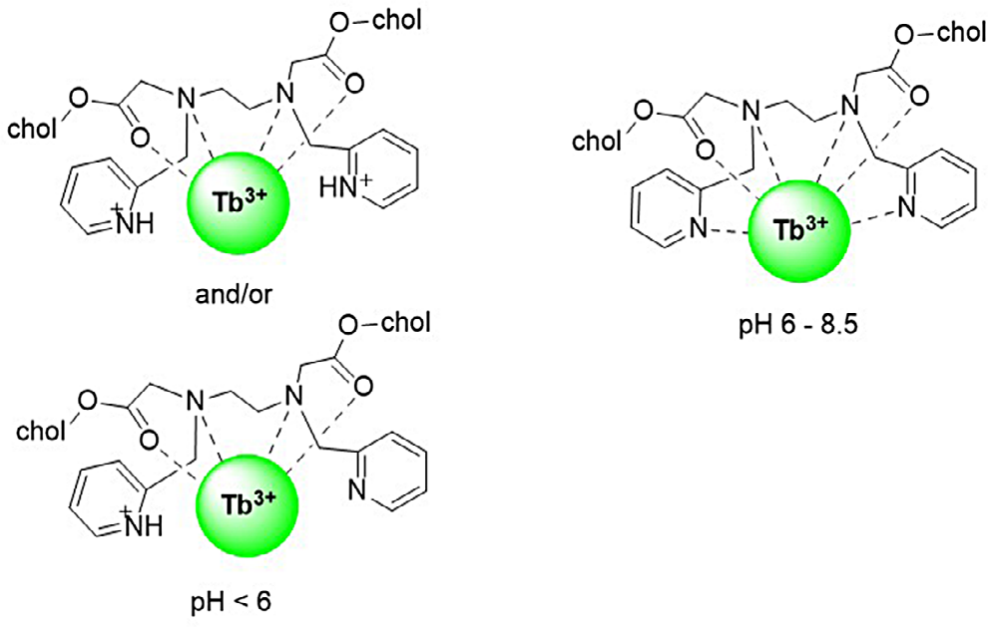
Possible coordination modes of the **L2**‐Tb complex.

Scheme 3 depicts possible structures of **L2**‐Tb depending on the pH value. Below pH = 6, one or both pyridine groups are protonated and located far from the metal center due to charge repulsion. In the pH range of 6–8.5, a hexacoordinated Tb^3+^ complex is formed. The pyridyl group effectively works as a sensitizer under weakly alkaline conditions, which suggests that the stability of self‐assembled **L2**‐Tb is relatively high despite its lower number of coordination sites. These features render self‐assembled **L2**‐Tb suitable for biological applications. The DLS measurements revealed no difference in the average particle size and distribution of self‐assembled **L2**‐Tb at various pH values (Figure S7). The particle size is comparable to that observed under the complexation conditions shown in Figure 4. This result indicates that **L2**‐Tb is stable under acidic and alkaline conditions, whereby the luminescence intensity depends on the protonation of the pyridine moiety. Interestingly, pH–responsive luminescence is observed even under the self‐assembling conditions. These results demonstrate the potential of self‐assembled **L2**‐Tb as a pH–dependent luminescence sensor in diverse fields, such as environmental analysis and bioanalytical chemistry.

**Figure 7:**
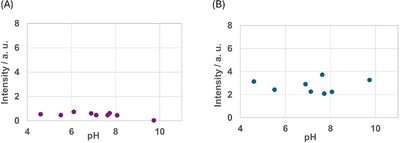
Luminescence intensity of a) self‐assembled **L**‐Tb and b) self‐assembled **L1**‐Tb at varying pH values. [**L**‐Tb] = [**L1**‐Tb] = 2.0 × 10^−5^ M in aqueous ethanol (20 wt.% EtOH); excitation wavelength (*λ*_ex_) = 230 nm.

As shown in Figure 7, the luminescence intensity of self‐assembled **L**‐Tb is almost the same in the pH range of 4–10, i.e., pH–dependent luminescence was not observed. Similarly, self–assembled **L1**–Tb showed no pH–dependent luminescence, most likely because **L1** prevents coordination of water to the metal center to a lesser extent than **L2** due to the lack of coordination sites.

The luminescence lifetimes were obtained for **L2**‐Tb complex (Table S2). The **L2**‐Tb in EtOH displays long luminescence lifetime at 1.68 ms, which is little shorter than that of tetrakis(2‐pyridylmethyl) ethylenediamine ligand (TPEN) Tb complex (TPEN–Tb(NO_3_)) in CH_3_CN (2.86 ms) reported by Nishihara et al.^[^ ^39^ ^]^ Ung et al. also reported the luminescence lifetime of TEPN–Tb(OTf)_3_ in CH_3_CN was 2.87 ms.^[^ ^40^ ^]^ The q value which indicate the number of coordinating water estimated for TPEN–Tb(NO_3_) in H_2_O is 0.2.^[^ ^39^ ^]^ The data for the luminescence lifetime of **L2**‐Tb indicate that few water molecules coordinated to the Tb ion. In assembled state, the luminescence lifetime of **L2**‐Tb in 20 wt.% EtOH was observed at 0.3 ms in both conditions of pH = 4.2 and pH = 8.5, respectively. These values were shorter than the TPEN–Tb(NO_3_) in H_2_O (τ = 1.33 ms, quantum yield Φ = 0.76), which also suggested that some water molecules coordinate to the Tb ion because of a few coordination site or weak interactions with pyridine units. From the results of lifetime analysis, the quantum yields of **L2**‐Tb may be expected to be high in EtOH solution. In assembled state, the quantum yields of **L2**‐Tb are expected to be approximately around 1/5 times in those of the nonassembled state.

## 3 Conclusion

Amphiphilic lanthanide complexes with ethylenediamine backbone ligands that bear two photoantenna groups were successfully synthesized, and their complexation and luminescence behavior in solution were investigated. ^1^H NMR titration experiments revealed that tetracoordinate ligand **L1** and hexacoordinate ligand **L2** form stable 1:1 complexes with Lu^3+^. **L2**‐Tb exhibited more intense luminescence in EtOH than **L**‐Tb and **L1**‐Tb, indicating that the pyridyl group effectively works as a photoantenna. DLS measurements showed that **L1**‐Tb and **L2**‐Tb form stable self‐assembled nanoparticles with a diameter of ∼40 nm in aqueous ethanol (20 wt.% EtOH), despite having only two cholesteryl substituents. In aqueous media, self‐assembled **L**‐Tb showed the strongest luminescence. We found that at least two hydrophobic cholesteryl groups are required to suppress the coordination of water effectively. Finally, pH–dependent studies revealed no change in the relative luminescence intensity of self‐assembled **L**‐Tb and **L1**‐Tb in the pH range of 4–10, whereas self‐assembled **L2**‐Tb exhibited pH–dependent luminescence. The unique behavior of **L2**‐Tb should most likely be attributed to the protonation of the pyridine moieties even in membrane. Based on the results of our study, we expect that the luminescent **L2**‐Tb complex with a flexible core find applications as a good host material for water soluble molecules.

## 4 Materials

Tb(OTf)_3_ (99.9%) and Lu(OTf)_3_ (99.9%) were purchased from Sigma Aldrich. Ethylenediamine was distilled from KOH before use. Cholesterol, *N*,*N*″–bis(phenylmethyl)‐1,2‐ethanediamine, and 2‐pyridinecarboxaldehyde were purchased from common commercial sources and used without purification. All other chemicals and solvents were of reagent grade and used as received. **L**,^[^ ^22^ ^]^ **L**‐Ln^3+^,^[^ ^22^ ^]^ 3β‐chloroacetoxy‐5‐cholestene,^[^ ^35^ ^]^ and *N*,*N*″–bis(2‐pyridylmethyl)‐1,2‐ethanediamine^[^ ^36^ ^]^ were synthesized according to reported procedures.

## 5 Experimental Section

### 5.1 Measurements

All spectroscopic measurements were performed at room temperature unless otherwise noted. ^1^H NMR (300 MHz) and ^13^C NMR (100 MHz) spectra were measured in CDCl_3_ with tetramethylsilane as the internal standard using a Bruker AVANCE 300 spectrometer at 293 K. Luminescence spectra were measured on a Perkin‐Elmer LS–55 fluorophotometer with a Xe flash lamp and a Hitachi F–4500 fluorophotometer. Absorption spectra were recorded on a JASCO V–670 spectrometer. High–resolution mass spectra (HRMS) were measured on JEOL AccuTOF LC–plus JMS–T100LP spectrometers. All melting points were measured by Yanaco micro melting points apparatus and are uncorrected. DLS measurements were performed on a Malvern Zetasizer Nano ZS, and the average of three independent measurements per sample is provided.

### 5.2 Synthesis

#### 3β–Chloroacetoxy–5–cholestene

Under a nitrogen atmosphere, a solution of triethylamine (3.50 g, 34.6 mmol) in dry CH_2_Cl_2_ (80 mL) was treated with cholesterol (9.60 g, 24.8 mmol). The mixture was cooled to 4 °C, before a solution of chloroacetyl chloride (2.84 g, 25.0 mmol) in CH_2_Cl_2_ (20 mL) was added dropwise. The reaction mixture was stirred for 22 h at rt, until TLC measurements on SiO_2_ (eluent, CHCl_3_) indicated completion of the reaction. The crude solution was then washed with water (80 mL), dried over MgSO_4_, and filtered, before the solvent was removed from the filtrate under reduced pressure. Purification by column chromatography (SiO_2_, CHCl_3_) afforded 3β‐chloroacetoxy‐5‐cholestene as a white solid (8.30 g, 17.9 mmol, 72%).

^1^H NMR (300 MHz, CDCl_3_) *δ* 0.69 (s, 3H), 0.80–2.10 (m, 38H), 2.36 (m, 2H), 4.03 (m,2H, COOCH_2_Cl), 4.69 (m, 1H, COOCH), 5.39 (m, 1H, C = CH).

#### N,N'–Bis(2–pyridinylmethylene)–1,2–ethanediamine

Ethylenediamine (0.90 g, 15 mmol) and 2‐pyridinecarboxaldehyde (3.16 g, 30 mmol) were dissolved in EtOH (4 mL), and the resulting solution was stirred for 5 h at rt. Evaporation of the solvent gave *N,N'*‐bis(2‐pyridinylmethylene)‐1,2‐ethanediamine as a brown oil (3.62 g, 15 mmol, >99%), which was used without further purification.

^1^H NMR (300 MHz, CDCl_3_) *δ* 4.06 (s, 4H), 7.30 (ddd, 2H, *J* = 7.5, 4.8, 1.2 Hz), 7.73 (td, 2H, *J* = 7.8, 1.8 Hz), 7.98 (dt, 2H, *J* = 7.8, 4.8 Hz), 8.42 (s, 4H), 8.62 (dq, 2H, *J* = 4.8 Hz).

#### N,N'–Bis(2–pyridylmethyl)–1,2–ethanediamine

An ice‐cooled suspension of *N*,*N*′–bis(2‐pyridinylmethylene)‐1,2‐ethanediamine (1.08 g, 4.50 mmol) in MeOH (8 mL) was treated with three portions of NaBH_4_ (each portion: 0.50 g; 1.50 g, 17.0 mmol). Then, the reaction mixture was allowed to warm to rt, before stirring was continued for 23 h. After the reaction reached completion, the solvent was removed under reduced pressure. The crude product was suspended in water (50 mL), extracted with CHCl_3_ (2 × 20 mL), and washed with water and brine. The organic layer was dried over MgSO_4_ and filtered, before the solvent was removed from the filtrate under reduced pressure. The product was obtained as a yellow oil (0.93 g, 3.8 mmol, 85%).

^1^H NMR (300 MHz, CDCl_3_) *δ* 2.82 (s, 4H), 3.92 (s, 4H), 7.13 (ddd, s, 2H, *J* = 7.5, 4.8, 1.2 Hz), 7.32 (d, 2H, *J* = 7.5 Hz), 7.62 (td, 2H, *J* = 7.5, 0.9 Hz), 8.54 (dm, 2H, *J* = 4.8 Hz).

#### N,N′–Dibenzyl–N,N′–acetylcholesteryl ethylenediamine (**L1**)

*N*,*N*′–Bis(phenylmethyl)‐1,2‐ethanenediamine (0.33 g, 1.36 mmol) and K_2_CO_3_ (0.37 g, 2.68 mol) were dissolved in CH_3_CN (5 mL) at rt. Under a nitrogen atmosphere, a solution of 3β‐chloroacetoxy‐5‐cholestene (1.04 g, 2.24 mol) in CHCl_3_ (4 mL) was added dropwise at rt. Then, the reaction mixture was stirred under reflux for 2 days. After cooling, the organic layer was washed with water, dried over MgSO_4_, and filtered, before the solvent was removed from the filtrate under reduced pressure. Purification by column chromatography (SiO_2_, *n*‐hexane:CHCl_3_ = 1:1) and GPC afforded **L1** as a white solid (0.86 g, 0.78 mmol, 70%).

L1: White Solid, m.p. 100–103 °c (decomp.): ^1^H NMR (300 MHz, CDCl_3_) *δ* 0.69 (s, 6H), 0.80–2.10 (m, 76H), 2.30 (m, 4H), 2.82 (s, 4H, NCH_2_CH_2_N), 3.33 (s, 4H, NCH_2_CO), 3.78 (s, 4H, NCH_2_Ph), 4.65 (m, 2H, COOCH) 5.37 (m, 2H, C = CH) 7.24–7.31 (m, 10H, C_6_ H_5_).

^13^C NMR (75 MHz, CDCl_3_) *δ* 12.00, 18.86, 19.45, 21.18, 22.70, 22.96, 23.97, 24.02, 24.42, 28.00, 28.15, 28.37, 31.99, 32.05, 35.95, 36.33, 36.64, 36.72, 37.41, 38.31, 39.65, 39.88, 42.46, 50.17, 51.64, 54.34, 56.32, 56.84, 58.66, 74.26, 122.86, 127.34, 128.43, 129.25, 138.59, 139.70, 170.83.

Anal. Calcd. for C_74_H_112_N_2_O_4_: C, 81.27; H, 10.32; N, 2.73, Found: C, 81.02 (+0.25%); H, 10.43 (−0.11%); N, 2.56 (−0.17%). HRMS (ESI–TOF, pos.): *m*/*z *= 1093.87047, calcd. for [C_74_H_112_N_2_O_4 _+ H]^+^: 1093.87003.

#### N,N′–Bis(2–pyridylmethyl)–N,N′–acetylcholesteryl ethylenediamine (**L2**)

Pyridinyl‐substituted ligand **L2** was synthesized following the same procedure as that for **L1**. Specifically, *N*,*N*′–bis(2‐pyridylmethyl)‐1,2‐ethanediamine (100 mg, 0.41 mmol) and K_2_CO_3_ (230 mg, 1.66 mmol) were dissolved in CH_3_CN (6 mL) at rt. Under a nitrogen atmosphere, a solution of 3β‐chloroacetoxy‐5‐cholestene (400 mg, 0.86 mmol) in CH_2_Cl_2_ (5 mL) was added dropwise at rt, before the reaction mixture was stirred under reflux for 2 days. After cooling, all volatiles were evaporated, and the residue was redissolved in CHCl_3_. The organic layer was washed with water, dried over MgSO_4_, and filtered, before the solvent was removed from the filtrate under reduced pressure. Purification of the residue by column chromatography (SiO_2_, *n*‐hexane:CHCl_3_ = 1:1) and GPC afforded **L2** as a white solid (260 mg, 0.24 mol, 59%).

L2: white solid, m.p. 104–105 °c (decomp.): ^1^H NMR (300 MHz, CDCl_3_) *δ* 0.69 (s, 6H), 0.80–2.10 (m, 76H), 2.30 (m, 4H), 2.85 (s, 4H, NCH_2_CH_2_N), 3.42 (s, 4H, NCH_2_CO), 3.93 (s, 4H, NCH_2_Py), 4.62 (m, 2H, COOCH), 5.37 (m, 2H, C = CH) 7.14 (dd, 2H, *J* = 7.5, 5.1 Hz), 7.47 (d, 2H, *J* = 7.5 Hz), 7.62 (dd, 2H, *J* = 7.5, 7.5 Hz), 8.51 (d, 2H, *J* = 5.1 Hz).

^13^C NMR (75 MHz, CDCl_3_) *δ* 11.94, 18.87, 19.40, 21.10, 22.67, 22.94, 23.90, 23.95, 24.36, 27.91, 28.10, 28.33, 31.89, 31.97, 35.90, 36.25, 36.62, 37.04, 38.21, 39.58, 39.79, 42.37, 50.05, 52.08, 55.34, 56.21, 56.75, 60.29, 74.35, 122.30, 122.84, 123.33, 136.91, 139.56, 148.88, 170.70. Anal. Calcd. for C_72_H_110_N_4_O_4_·H_2_O: C, 77.65; H, 10.14; N, 5.03, Found: C, 77.48 (−0.17%); H, 10.08 (−0.06%) N, 5.16 (+0.13%). HRMS (ESI–TOF, pos.): *m*/*z *= 1095.85576, calcd. for [C_74_H_111_N_4_O_4 _+ H]^+^: 1095.86053.

#### Preparation of Self–assembled Tb^3+^ Complexes

A solution of the Tb^3+^ complex (**L**‐Tb, **L1**‐Tb, or **L2**‐Tb) in aqueous ethanol (20 wt.% EtOH) containing bis–tris buffer (pH = 7.0, bis–tris (HCl)) was prepared by adding the buffered aqueous solution (1.7 × 10^−3^ M) to an EtOH solution of the Tb^3+^ complex. In the case of pH–titration experiments, ultrapure water was used instead of bis–tris buffer, and 0.1 M KOH aq. or 0.1 M HCl aq. was used for pH adjustment. DLS measurements and spectroscopic measurements were conducted at least 1 h after the solution was prepared to ensure equilibrium conditions.

## Supporting Information

The data that support the findings of this study are available in the Supporting Information of this article.

## Conflict of Interest

The authors declare no conflict of interest.

## Supporting information



Supporting Information

## Data Availability

The data that support the findings of this study are available in the supplementary material of this article.
