# Supplementary material for: pH–Dependent Luminescence of Self–Assembly Tb3+ Complexes with Photosensitizing Units
Source: Chemistry. 2025 Sep 19;31(62):e02378. doi: 10.1002/chem.202502378 (PMC12598380; doi:10.1002/chem.202502378)
Supplement: Supplementary file 1 — Supporting Information [file CHEM-31-e02378-s001.pdf]

# Supporting Information

## pH-Dependent Luminescence of Self-Assembly $Tb^{3+}$ Complexes with Photosensitizing Units

Eiko Mieda,* Tatsuya Watanabe, Ryusei Morita, Hiroyuki Miyake, and Satoshi Shinoda*

Department of Chemistry, Graduate School of Science, Osaka Metropolitan University,
3-3-138 Sugimoto, Sumiyoshi-ku, Osaka 558-8585, Japan

*E-mail: mieda@omu.ac.jp, shinoda-s@omu.ac.jp

**Contents**

・**$^{1}$H and $^{13}$C NMR spectra of Ligand** (Figure S1−S4)

・**Absorption spectra** (Figure S5, S6)

・**DLS measurement** (Figure S7, Table S1)

・**Luminescence spectra** (Figure S8, S9)

・**Luminescence properties** (Table S1−S3, Figure S10, S11)

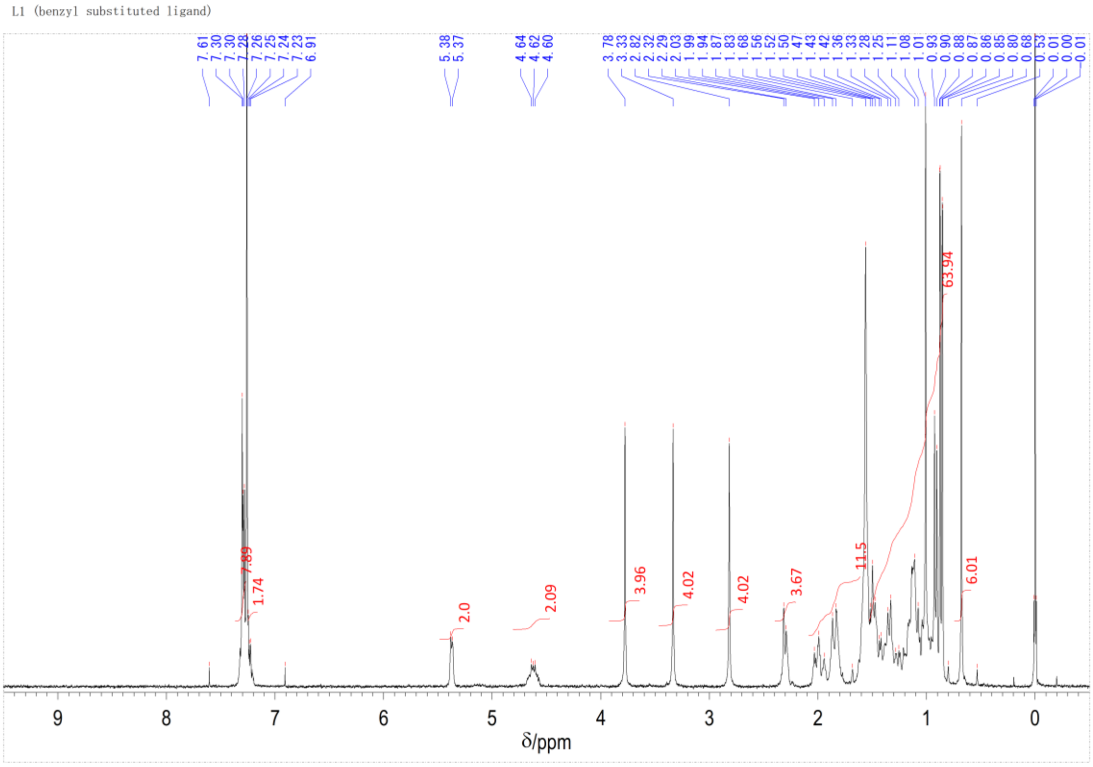


Figure S1 $^{1}$H NMR spectrum of **L1** in $CDCl_3$

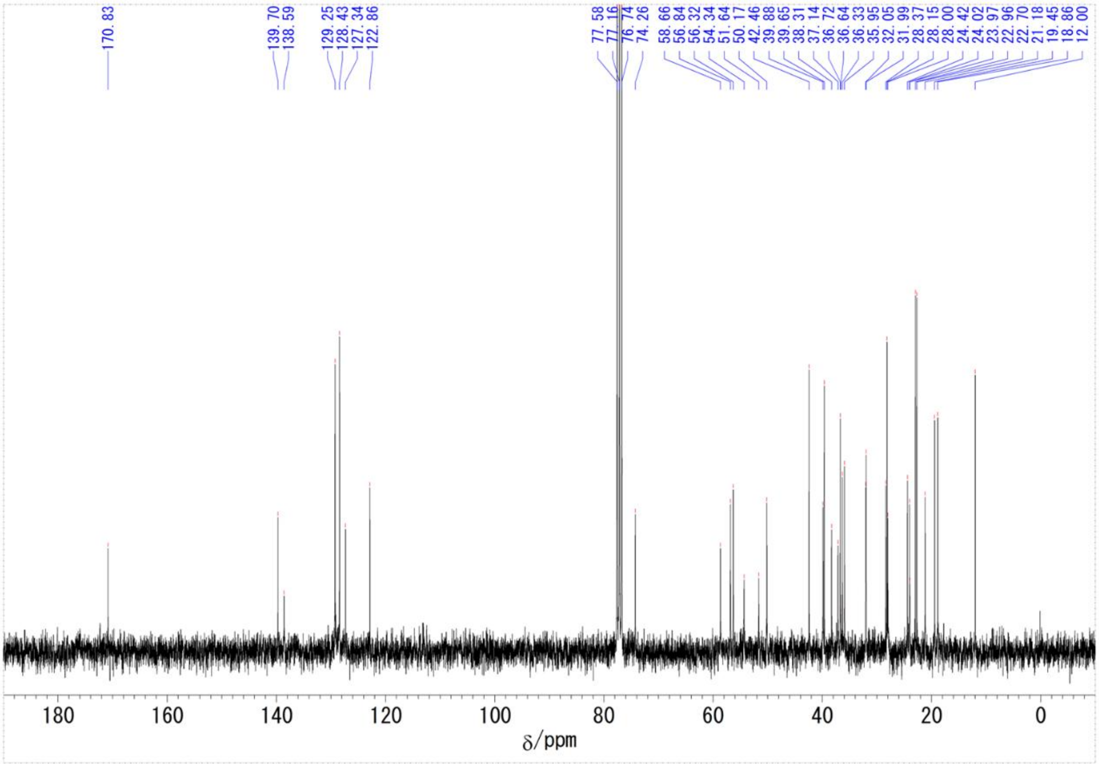


Figure S2 $^{13}$C NMR spectrum of **L1** in $CDCl_3$

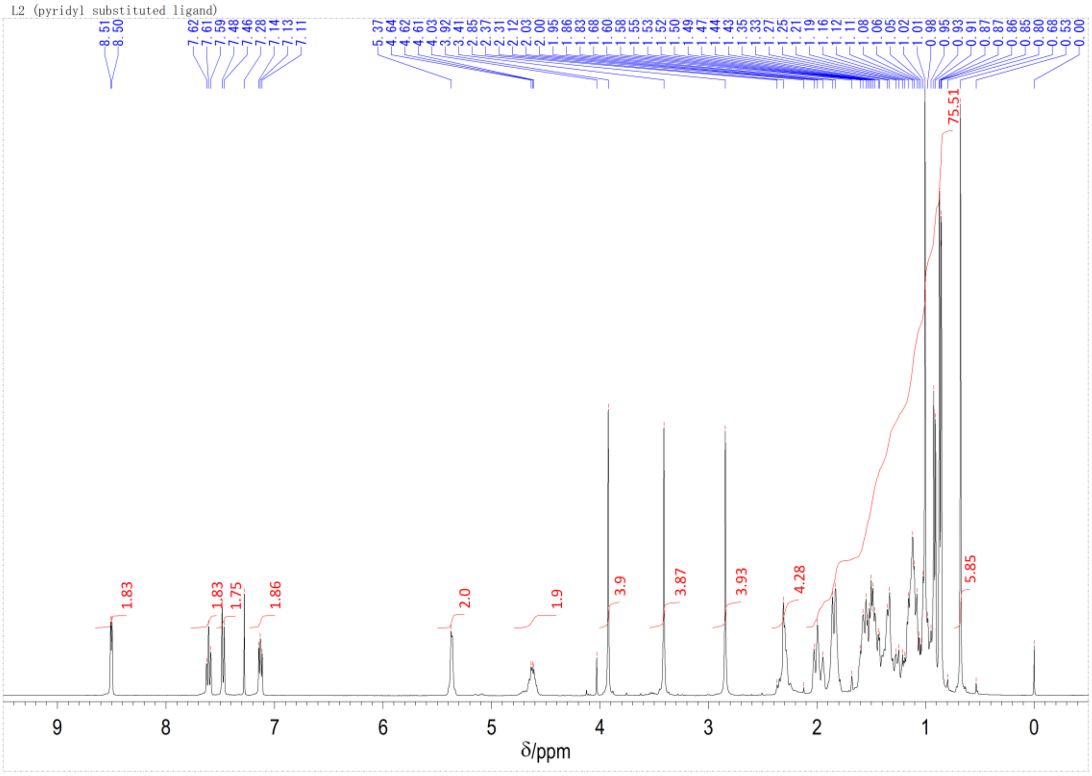


Figure S3 $^{1}$H NMR spectrum of **L2** in $CDCl_3$

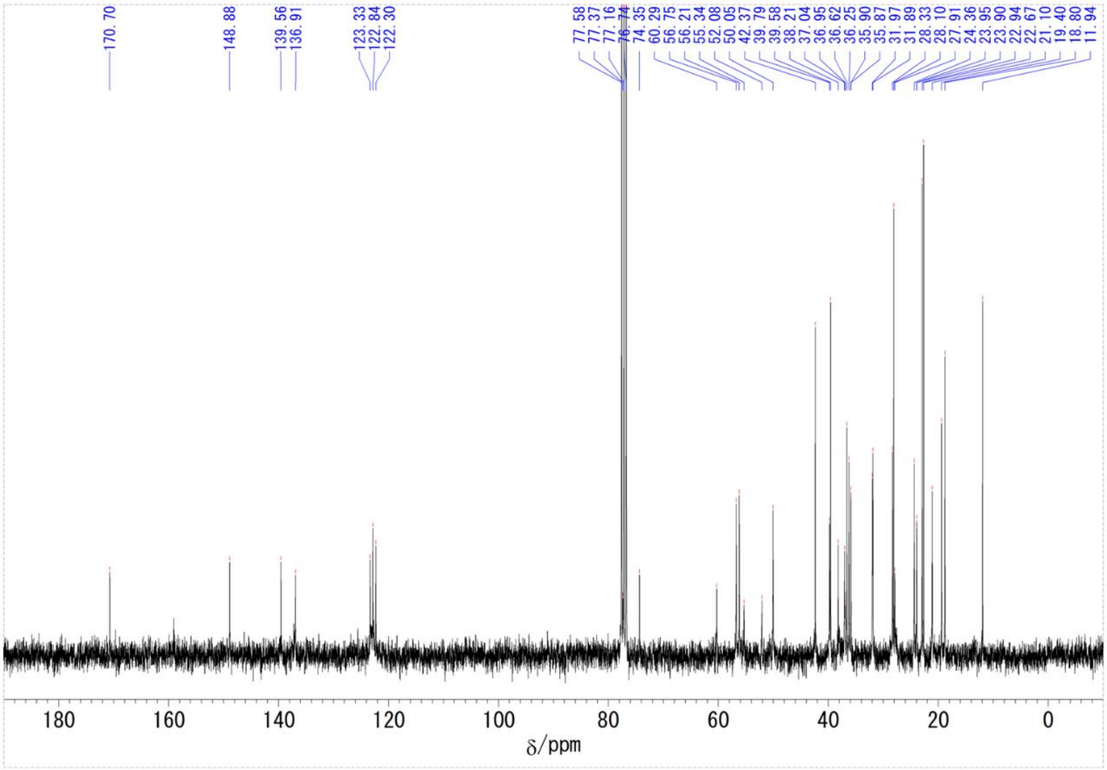


Figure S4 $^{13}$C NMR spectrum of **L2** in $CDCl_3$

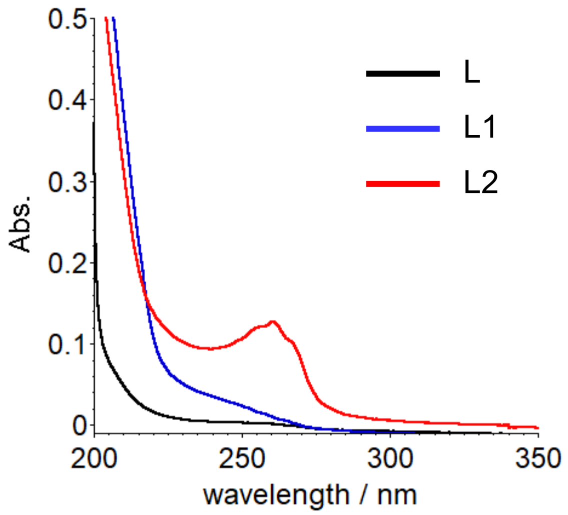


Figure S5 UV-vis spectra of **L**, **L1** and **L2**. [**L**] = [**L1**] = [**L2**] = $2.0 \times 10^{-5}$ M in EtOH.

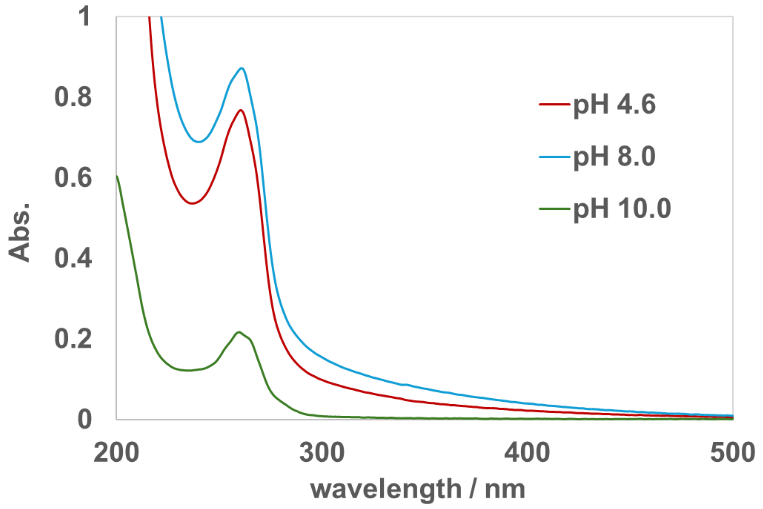


Figure S6 UV-vis spectra of **L2**-Tb assembly under various pH conditions. [**L2**-Tb] = $1.0 \times 10^{-4}$ M in 20 wt% EtOH/$H_2O$. 0.1 M KOH aq. or 0.1 M HCl aq. was used for pH adjustment.

(a) (b)

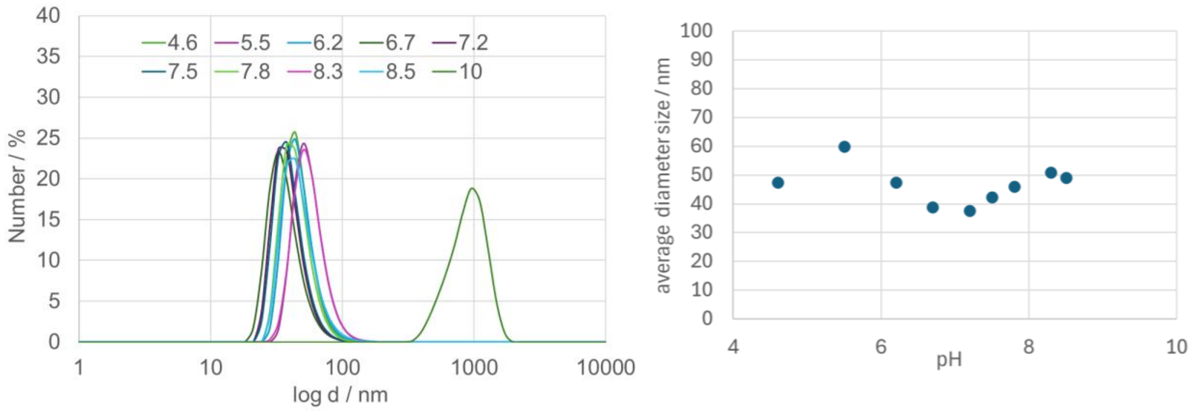


Table S1

| pH | average diameter size / nm | Std. / nm |
|---|---|---|
| 4.6 | 48 | 12.4 |
| 5.5 | 60 | 16.1 |
| 6.2 | 47 | 13.6 |
| 6.7 | 39 | 11.4 |
| 7.2 | 38 | 10.9 |
| 7.5 | 42 | 11.6 |
| 7.8 | 46 | 12.5 |
| 8.3 | 51 | 15.8 |
| 8.5 | 49 | 15.3 |
| 10 | 765.5* | 166.8 |

*Precipitate was formed.

Figure S7 (a) Particle size distribution (DLS) of **L2**-Tb under various pH conditions. (b) Size difference of these conditions. These average size and standard deviations are listed in the Table S1. [**L2**-Tb] = $1.0 \times 10^{-4}$ M in 20 wt% EtOH/$H_2O$.

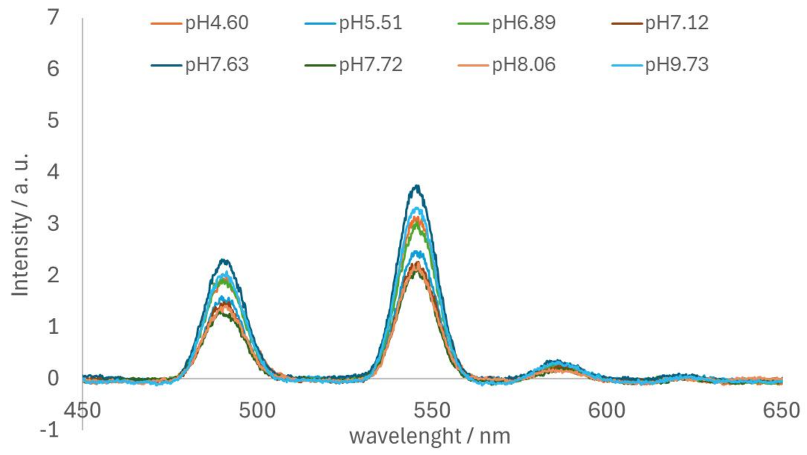


Figure S8 Luminescence spectra of **L**-Tb assembly under various pH conditions. [**L**-Tb] = 2.0 × $10^{-5}$ M, 20 wt% EtOH/$H_2O$. Excitaion wavelength ($\lambda_{ex}$) = 230 nm.

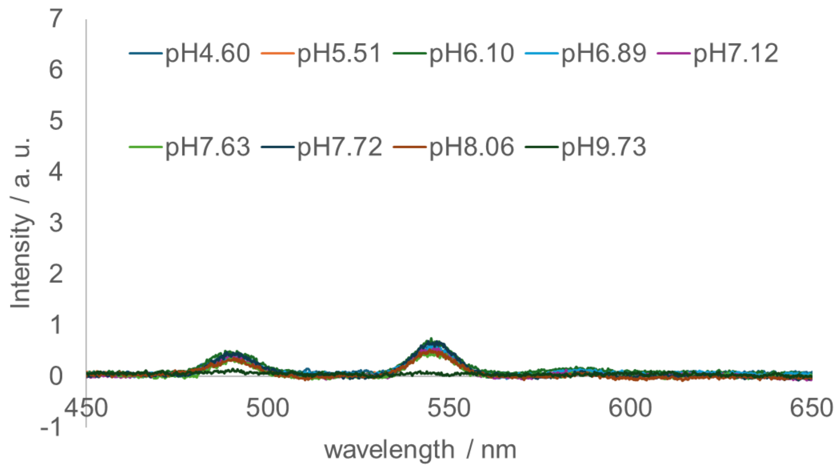


Figure S9 Luminescence spectra of **L1**-Tb assembly under various pH conditions. [**L1**-Tb] = 2.0 × $10^{-5}$ M, 20 wt% EtOH/$H_2O$. Excitation wavelength ($\lambda_{ex}$) = 230 nm

Table S2 Luminescence lifetime of **L2**-Tb. Conditions: (a) [**L2**-Tb]=1.0×$10^{-4}$ M in EtOH, (b) and (c) 20 wt% EtOH aqueous solution. Excitation wavelength ($\lambda_{ex}$) = 260 nm.

| sample | solvent | Lifetime [ms] |
|---|---|---|
| (a) | EtOH | 1.680 |
| (b) | 20 wt% EtOH/$H_2O$, pH=8.5 | 0.316 |
| (c) | 20 wt% EtOH/$H_2O$, pH=4.2 | 0.321 |

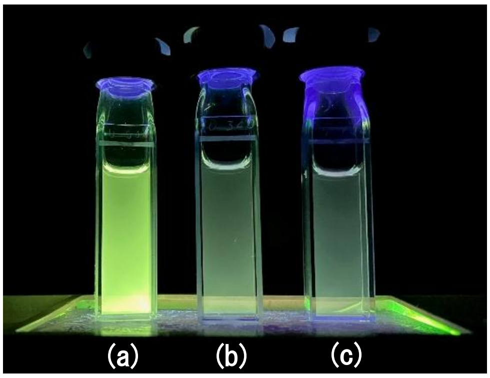


Figure S10 Photograph of the **L2**-Tb under UV light (256 nm), (a) [**L2**-Tb]=1.0×10$^{-4}$ M in EtOH, (b) [**L2**-Tb]=1.0×10$^{-4}$ M in 20 wt% EtOH/$H_2O$, pH=8.5, (c) [**L2**-Tb]=1.0×10$^{-4}$ M in 20 wt% EtOH/$H_2O$, pH=4.2.

Table S3 Data varidation of luminescence intensity of **L2**-Tb assembly. The intensity at 546 nm was used for the analysis. [**L2**-Tb]=1.0×10$^{-4}$ M in 20 wt% EtOH/$H_2O$. Excitaion wavelength ($\lambda_{ex}$) = 260 nm.

| **pH** | 4.16[*1] | 8.53[*1] | 7.02[*1] | 7.02[*2] |
|---|---|---|---|---|
| | 0.891 | 1.395 | 0.999 | 0.999 |
| | 0.855 | 1.444 | 0.936 | 0.962 |
| | 0.796 | 1.427 | 0.989 | 1.164 |
| average | 0.847 | 1.422 | 0.975 | 1.042 |
| Std. | 0.039 | 0.020 | 0.028 | 0.088 |

[*1] The values of 3 times scan for a sample were evaluated.

[*2] The values of measurements from 3 samples were evaluated.

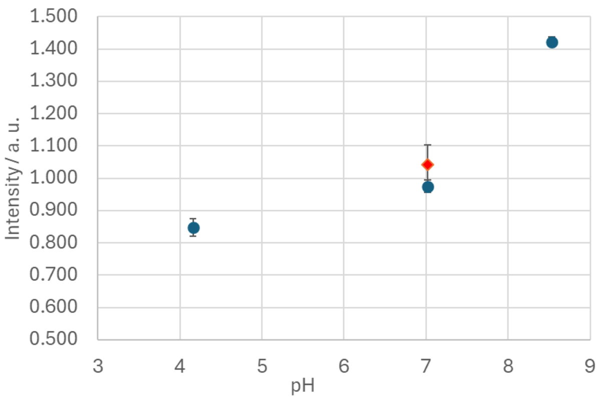


Figure S11 Plots of the average intensity of **L2**-Tb assembly at 546 nm. (The conditions and values were listed in Table S3)
